# Differences in spontaneous speech fluency between Parkinson's disease and spinocerebellar ataxia type 3

**DOI:** 10.3389/fneur.2023.1179287

**Published:** 2023-05-05

**Authors:** Vanessa Brzoskowski dos Santos, Annelise Ayres, Maiara Laís Mallmann Kieling, Elaine Cristina Miglorini, Laura Bannach Jardim, Artur Francisco Schumacher-Schuh, Carlos Roberto de Mello Rieder, Raphael Machado de Castilhos, Kristie Spencer, Rui Rothe-Neves, Maira Rozenfeld Olchik

**Affiliations:** ^1^Post-graduate Program in Medicine: Medical Sciences, Universidade Federal do Rio Grande do Sul, Porto Alegre, RS, Brazil; ^2^Neurology Service, Hospital de Clínicas de Porto Alegre, Porto Alegre, RS, Brazil; ^3^Post-graduate Program in Genetics and Molecular Biology, Universidade Federal do Rio Grande do Sul, Porto Alegre, RS, Brazil; ^4^Medical Genetics Service, Hospital de Clínicas de Porto Alegre, Porto Alegre, RS, Brazil; ^5^Department of Internal Medicine, University of Medicine, Universidade Federal do Rio Grande do Sul, Porto Alegre, RS, Brazil; ^6^Department of Pharmacology, Universidade Federal do Rio Grande do Sul, Porto Alegre, RS, Brazil; ^7^Post-Graduate Program in Rehabilitation Sciences, Universidade Federal de Ciências da Saúde de Porto Alegre, Porto Alegre, RS, Brazil; ^8^Department of Speech and Hearing Sciences, University of Washington, Seattle, WA, United States; ^9^Phonetics Laboratory of the Faculty of Letters, Universidade Federal de Minas Gerais, Belo Horizonte, MG, Brazil; ^10^Department of Surgery and Orthopedics, Faculdade de Odontologia, Universidade Federal do Rio Grande do Sul, Porto Alegre, RS, Brazil

**Keywords:** Parkinson's disease, spinocerebellar ataxia, speech disorders, dysarthria, articulation disorders

## Abstract

**Background:**

The basal ganglia and cerebellum both have a role in speech production although the effect of isolated involvement of these structures on speech fluency remains unclear.

**Objective:**

The study aimed to assess the differences in the articulatory pattern in patients with cerebellar vs. basal ganglia disorders.

**Methods:**

A total of 20 individuals with Parkinson's disease (PD), 20 with spinocerebellar ataxia type 3 (SCA3), and 40 controls (control group, CG) were included. Diadochokinesis (DDK) and monolog tasks were collected.

**Results:**

The only variable that distinguished SCA3 carriers from the CG was the number of syllables in the monolog, with SCA3 patients of a significantly lower number. For patients with PD, the number of syllables, phonation time, DDK, and monolog were significantly lower than for CG. Patients with PD were significantly worse compared to patients with SCA3 in the number of syllables and phonation time in DDK, and phonation time in monolog. Additionally, there was a significant correlation between the number of syllables in the monolog and the MDS-UPDRS III for participants with PD, and the Friedreich Ataxia Rating Scale for participants with SCA3 suggesting a relationship between speech and general motor functioning.

**Conclusion:**

The monolog task is better at discriminating individuals with cerebellar vs. Parkinson's diseases as well as differentiating healthy control and was related to the severity of the disease.

## Introduction

Spinocerebellar ataxia type 3 (SCA3), also known as Machado–Joseph disease (MJD), is an autosomal dominant neurodegenerative disease, caused by the expansion of a repetitive CAG sequence in the ATXN3 gene located on chromosome 14q32 (1, 2). The prevalence of SCAs has been estimated to be two cases per 1,00,000 individuals worldwide; among these, MJD is known as the most frequent SCA in the world. In Rio Grande do Sul and Portugal, the minimum estimated prevalence is 3 cases per 1,00,000 inhabitants (3, 4). Speech disorders in SCA3 are characterized as ataxic dysarthria. The main characteristics of ataxic dysarthria, related to the disruption of the cerebellar circuit, are articulatory imprecision, prosodic excess, variable stress patterns, prolonged phonemes and pauses (leading to “scanning” speech), and phonatory-prosodic insufficiency (5–7).

Parkinson's disease (PD) is a neurodegenerative disorder that affects both motor and non-motor systems. While it was previously thought to be a disease of the basal ganglia, current research suggests that it may also involve other regions of the brain, including the cortex and brainstem (8). PD is the second most prevalent neurodegenerative disease in the world, with a worldwide incidence of between 1 and 20 per 1,000 individuals/year (9). Although the motor symptoms of tremor, bradykinesia, and rigidity are more prevalent, there is also a high incidence of dysarthria in this population. Dysarthria in PD is of the hypokinetic type, characterized by a reduction in the speed and amplitude of movements that are necessary for speech, resulting in a monotonous, breathy, and soft speaking voice. Hypokinetic dysarthria can also cause imprecise articulation, resulting in slurred speech, and difficulty in initiating and terminating speech (5–7, 10).

Diadochokinesis (DDK) is a maximum-performance speech function that has been shown to differentiate between distinct neurological conditions (6, 7, 11, 12). DDK taxes the speech system, requiring maximum articulator speed, precision, and coordination, with a minimal cognitive-linguistic load. Spontaneous speech, on the other hand, requires cognitive-linguistic processing in addition to speech subsystem coordination (6, 7, 11). As the execution of movements for speech production relies heavily on subcortical (and cortical) components (13), perhaps some speech tasks can help to differentiate the motor control of speech for two subcortical diseases (PD and SCA), as suggested, for instance, by Ziegler (14). Although dysarthria in PD and SCA3 is theoretically well-defined, less is known about the differentiation of specific articulatory parameters as well as the relationship to overall motor function. Therefore, the study of these parameters can be helpful for understanding these conditions and developing more effective therapeutic strategies.

This study aimed to assess whether there are tasks that differentiate the articulatory pattern in patients with spinocerebellar ataxia type 3 (SCA3) (a cerebellar disease) and Parkinson's disease (PD) (a basal ganglia disease) compared with controls (CG).

## Materials and methods

A cross-sectional study was carried out at a university hospital in Porto Alegre, Brazil. Patients were recruited from the movement disorders outpatient clinics of Neurology and Medical Genetics Services from January to December 2021. The sampling process was convenience sampling. All patients treated at the service during the research period were invited to participate in the study. Individuals with a clinical diagnosis of Parkinson's disease (PD) or a genetic diagnosis of SCA3 were included. Individuals were excluded for systemic conditions or other neurological disorders that could affect speech patterns, as well as severe hearing loss. Healthy individuals, unrelated to the speakers and matched by age, were recruited from the community as a control group.

Informed consent was obtained from all subjects prior to any study procedure. The Research Ethics Committee approved the project by number 2019-0789. A preliminary version was presented in PAS-MDS 2022, poster number 49.

All procedures were performed by evaluators trained to apply speech tasks. Subjects from all groups were evaluated in a quiet room. The total duration of evaluation for each individual was approximately 10 min. The collection of speech samples consisted of recording tasks that assess the articulatory subsystem. Participants were asked to perform the diadochokinesis (DDK) task based on the alternating repetition of syllables [pataka] as fast as the individual could in a single breath and spontaneous speech based on the question “How was your day?” for 60 s. The recording was performed with Audacity software version 2.3.2 and a KARSECT HT-9 microphone, positioned approximately 5 cm from the subject's lips, which was coupled to the Andrea Pureaudio USB adapter and connected to a computer which sampled the voice signals at 44.1 kHz with 16-bit resolution (12).

Subsequently, all audio files were edited and normalized using the Praat program (15) version 5.1. We used a script to detect syllables from the loudness peaks and measure the speech rate automatically with Praat. As Brazilian Portuguese only allows for vowels at the syllable nucleus, counting the intensity peaks equals determining the number of syllables. The analyzed parameters were based on Rusz et al. (16) and Vogel and Maruff (17). The following dependent variables were analyzed for the monolog and DDK: (1) number of syllables, (2) phonation time, representing when a continuous speech signal was interrupted, and (3) articulation rate, which reflects the density of speech delivery (16, 17).

In addition, sociodemographic data (age, sex, and disease duration) and neurological severity data were collected from specific scales applied to each of the neurodegenerative diseases as follows: Movement disorder society-sponsored revision of the unified Parkinson's disease rating Scale (MDS-UPDRS) (18) part III and the activities of daily living subscale from the Friedreich Ataxia Rating Scale (FARS-ADL) (19).

Independent variables were presented as descriptive analyses (absolute and relative frequencies, mean and standard deviation). ANOVA with Bonferroni's test was used for the acoustic analysis of the articulation variables between the groups (SCA3 × PD; SCA3 × CG; PD × CG). Statistical significance was defined as a *p* < 0.05. The statistical software used was SPSS version 22.0.

## Results

**Figure 1:**
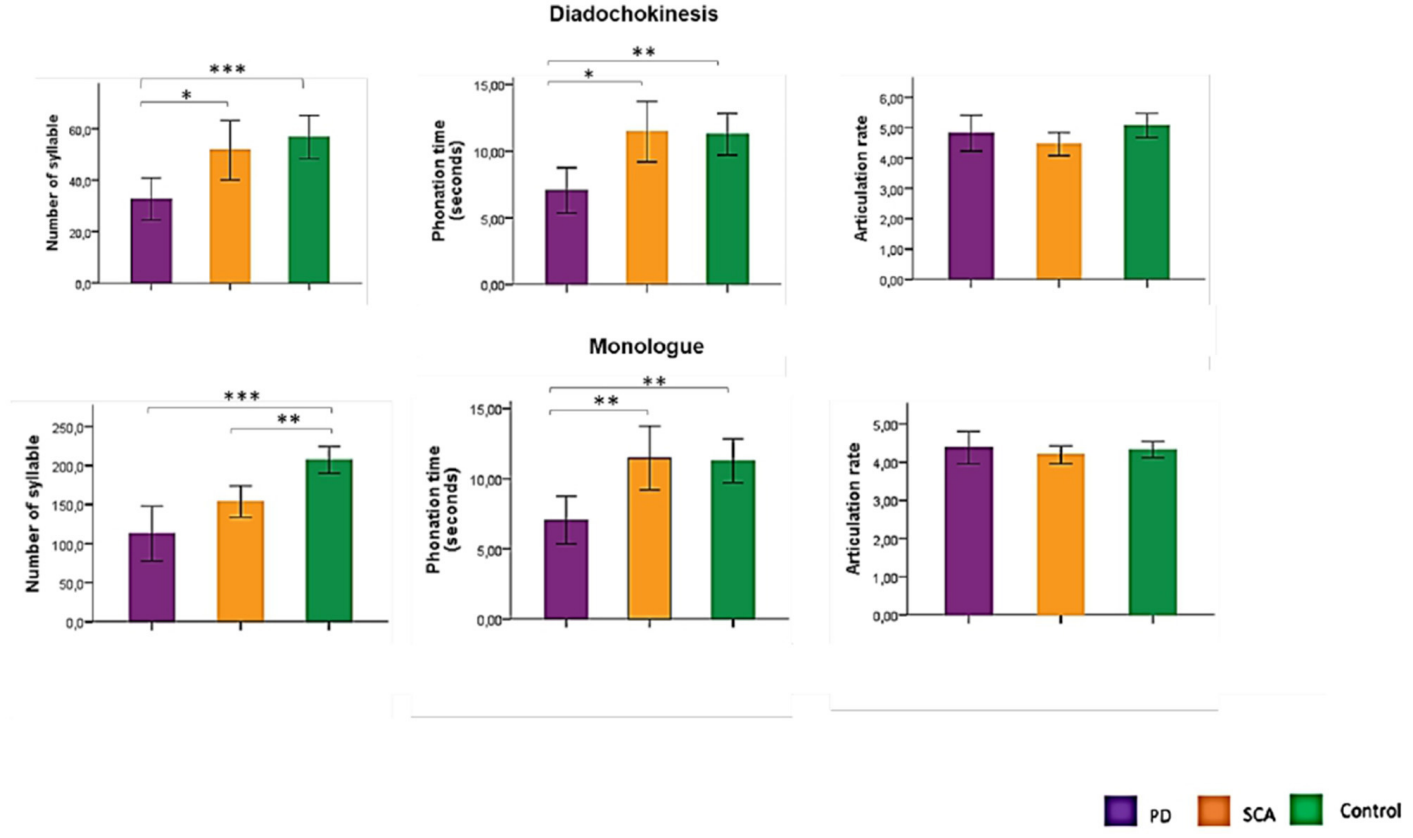
Comparison between groups in the acoustic analysis of diadochokinesis and monolog variable. PD, Parkinson's disease; SCA3, Spinocerebellar ataxia type 3; ^*^p = 0.05; ^**^p = 0.01; ^***^p < 0.0001. ANOVA with Bonferroni's test.

Table 1 presents the sociodemographic and clinical data of the sample. There was no statistical difference between groups regarding age and gender. As shown in Figure 1, there are DDK differences between PD and SCA3 in the number of syllables and phonation time (*p* = 0.05) and monolog differences between PD and SCA3 for phonation time (*p* = 0.01). The only variable that distinguished SCA3 carriers from controls was the number of syllables in the monolog, with SCA3 patients having a significantly lower number. For patients with PD, the number of syllables, and phonation time in DDK, and monolog were significantly lower than in controls.

**Table 1:** Sociodemographic data.

	**PD (*n* = 20)**	**SCA3/MDJ (*n* = 20)**	**Control (*n* = 40)**	***p*-value**
Male	15 (75%)	7 (35%)	15 (37%)	0.072
Age (years)	53.45 (±6.13)	47.6 (±10.46)	50.0 (±9.48)	0.130
Disease duration (years)	13.01 (±6.02)	10.95 (±6.17)	-	-
Education	7.75 (±1.81)	10.80 (±4.11)		
MDS/UPDRS-III	18.35 (±8.14)	-	-	-
H&Y	1.1 (±1.14)	-		
FARS-ADL	-	17.17 (±7.11)	-	-

PD, Parkinson's disease; SCA3, Spinocerebellar ataxia type 3; MDS/UPDRS-III, Movement disorder society-sponsored revision of the unified Parkinson's disease rating Scale part III; FARS-ADL, Friedreich Ataxia Rating Scale.

The number of syllables in the monolog was significantly correlated with the UPDRS (*p* = 0.039; *r* = −0.464) among PD subjects and with the FARS (*p* = 0.017; *r* = −0,527) among SCA3 subjects.

## Discussion

The spontaneous speech task (monolog) was able to differentiate the articulatory pattern in patients with SCA3 and PD. PD subjects spoke and articulated less compared with SCA3 and the control group. The monolog is a sensitive and simple task, regardless of education, to help detect speech disorders. Although the DDK task is very commonly used (6, 7, 12, 16, 17), it was altered only in PD patients and was not sensitive to differences between the SCA3 and control groups.

Additionally, it was observed that eleven patients (55%) from the SCA3 group had changes in diadochokinesis production, three had a slower production in the execution of the task, and six had a standard performance. The most frequently observed errors included changes in syllable order (/PATAKA/ for /KAPATA/ and /TAPAKA/). These findings corroborate the literature, which found production irregularities and distortions in the production of DDK in subjects with SCA3 (20, 21). However, those errors did not imply a fluency breakdown as much as reflected in the measurements used here. As a result, DDK was not sensitive for differentiating controls from patients with SCA3 in our sample. There is some suggestion that alternation motion rates (/PAPAPA/) are able to detect issues with speech motor control in cerebellar diseases more than sequential motion rates (/PATAKA/) (22).

The presence of errors and distortions in the DDK in patients with SCA3 shows the function of the cerebellum in speech production, which tunes the coordination of rapid movements and controls movement. As demonstrated in the literature (13, 20, 23), the cerebellum contributes to sensorimotor adaptation and is associated in neuroimaging studies of motor learning, with the size or frequency of sensory error. It sends corrective motor commands and contributes to feedback-based motor learning. The role of the basal ganglia is linked to motor functioning, mainly in the programming of the movement and in its learning, which can also involve other brain regions, including the cortex and brainstem (14).

Among their functions, the basal ganglia have regulatory action concerning speech. Changes to that regulation alter the ability to control movement in a dynamic and coordinated way. Reduction in the number of syllables and phonation time in patients with PD is described in the literature as a function of the basal ganglia in speech production and can reflect bradykinesia and difficulty with motor initiation. This region is involved in the regulation of voluntary movements (including initiation, speed, range, and direction), automatic movements (such as breathing movements), balance and posture movements, facial movements (such as facial expressions), and eye movements (5, 24, 25).

It must be noted, however, that disorders observed in patients with cerebellar disease are generally mild and test scores frequently fall into the lower end of the normal range (20). Among the tasks used, DDK proved to be a promising variable for discriminating patients with PD from patients with SCA3 although it was impossible to distinguish SCA3 from control subjects at the DDK task in our study; only the PD patients differed from controls.

There are articulatory differences when producing a syllable (consonant-vowel) inserted in a monolog and the production of diadochokinesis. Initially, DDK requires the standardized repetition of the same cycle of movements, unlike spontaneous speech, which involves a sequencing of different systems. In addition, DDK is a maximum performance task and requires a maximum rate of syllables with a consistent rhythm, whereas, in a monolog, the syllable rate is habitual and may reflect cognitive-linguistic load. Thus, these two motor tasks imply different physiological mechanisms and are differentially susceptible to the consequences of brain injury (14, 26).

Interestingly, SCA3 patients were slightly slower than the other groups as reflected by the articulation rate (syllables per second) although this difference was not statistically significant—a fact that may be attributed to a rather small and heterogeneous sample. Nevertheless, SCA3 patients took more time to produce fewer syllables than the others in the monolog, a condition where the maximum performance requirement does not apply. It is the pattern of differences across both measures (phonation time and the number of syllables), coupled with the trend in articulation rate, that highlights the crucial differences between the groups.

The level of education influences the performance of subjects in tests involving language. In low-income countries, such as Brazil, there is a disparity in opportunities for access to formal education, with a high proportion of individuals who did not attend a school or attended for a period shorter than 4 years (27). Thus, the use of tasks that involve reading skills can provide bias as the patient may present an increase in the number of pauses caused by the reading difficulty and not only by the change in the articulatory pattern.

Although the guideline for speech recording and acoustic analyses (12) recommends the use of sequential, alternating motion rates, reading passage, and monolog as integral aspects of motor speech assessment in low-income countries, a large proportion of patients have low education. Therefore, text-reading tasks become more error-prone and practically unfeasible. The finding that the monolog showed to be an efficient measure to differentiate patients with PD vs. SCA3 vs. controls recommends this task for populations with different equities rather than the use of reading tasks (27).

The identification of a specific task helps in clinical practice, contributing to diagnosis and speech therapy. Thus, it is suggested that the collection of speech samples from a monolog and the extraction of data related to the number of syllables and phonation time should be included in a minimum protocol for articulatory assessment in patients with PD and SCA3. Automatic extraction in PRAAT software is efficient and overcomes the challenges with perceptual judgments in speakers with reduced intelligibility. Repetition errors in the DDK, such as a change in the syllable order, maybe another important source of information as SCA patients may perform well as related to the number of syllables produced.

In conclusion, using a simple question for monolog collection proved to be the best task to discriminate the articulatory pattern between patients with cerebellar vs. Parkinson's diseases and healthy subjects. In addition, both in SCA3 and PD, the monolog was correlated with disease severity in their respective scales, the lower the production of syllables in the speech, the greater the severity of the disease. Whether or not this pattern of results extends to patients with other cerebellar vs. basal-ganglia diseases is still an open question.

## Data availability statement

The original contributions presented in the study are included in the article/supplementary material, further inquiries can be directed to the corresponding author.

## Ethics statement

The studies involving human participants were reviewed and approved by Research Ethics Committee of Hospital de Clínicas de Porto Alegre, Porto Alegre, Brazil, number 2019-0789. The patients/participants provided their written informed consent to participate in this study.

## Author contributions

VS: statistical analysis—conception, organization, and execution, statistical analysis—design and execution, and manuscript preparation—writing of the first draft and review and critique. AS-S, LJ, KS, RC, and CR: statistical analysis—review and critique and manuscript preparation—review and critique. EM and MK: research project—organization and review and critique. MO: research project—conception, organization, and execution and manuscript preparation–writing of the first draft and review and critique. RR-N: research project—conception, statistical analysis—design, execution, and review and critique, and manuscript preparation—review and critique. All authors contributed to the article and approved the submitted version.

## References

[1] Li T Martins S Peng Y Wang P Hou X Chen Z. Is the high frequency of machado-joseph disease in China due to new mutational origins? Front Genet. (2019) 9:740. 10.3389/fgene.2018.00740 30842792 PMC6391318

[2] Prestes PR Saraiva-Pereira ML Silveira I Sequeiros J Jardim LB. Machado-Joseph disease enhances genetic fitness: a comparison between affected and unaffected women and between MJD and the general population. Ann Hum Genet. (2008) 72:57–64. 10.1111/j.1469-1809.2007.00388.x 17683516

[3] de Castilhos RM Furtado GV Gheno TC Schaeffer P Russo A Barsottini O. Rede Neurogenetica. Spinocerebellar ataxias in Brazil-frequencies and modulating effects of related genes. Cerebellum. (2014) 13:17–28. 10.1007/s12311-013-0510-y 23943520

[4] Coutinho P Ruano L Loureiro JL Cruz VT Barros J Tuna A. Silva MC.Hereditary ataxia and spastic paraplegia in Portugal: a population-based prevalence study. JAMA Neurol. (2013) 70:746–55. 10.1001/jamaneurol.2013.1707 23609960

[5] Darley FL Aronson AE Brown JR. Differential diagnostic patterns of dysarthria. J Speech Hear Res. (1969) 12:246–69. 10.1044/jshr.1202.246 5808852

[6] Duffy JR. BC-ANCDS. Motor Speech Disorders: Substrates, Differential Diagnosis, and Management. Amsterdam: Elsevier Health Sciences. (2020).

[7] Kent RD KJF. Task-based profiles of the dysarthrias. Folia Phoniatr Logop. (2000) 52:48–53. 10.1159/000021512 10474004

[8] Marsili L Rizzo G Colosimo C. Diagnostic criteria for parkinson's disease: from James Parkinson to the concept of prodromal disease. Front Neurol. (2018) 9:156. 10.3389/fneur.2018.00156 29628907 PMC5877503

[9] Van Den Eeden SK Tanner CM Bernstein AL Fross RD Leimpeter A Bloch DA. Incidence of Parkinson's disease: variation by age, gender, and race/ethnicity. Am J Epidemiol. (2013) 157:1015–22. 10.1093/aje/kwg068 12777365

[10] Skodda S Schlegel U. Speech rate and rhythm in Parkinson's disease. Mov Disord. (2008) 23:985–92. 10.1002/mds.21996 18383114

[11] Van der Graaff M Kuiper T Zwinderman A Van de Warrenburg B Poels P Offeringa A. Clinical identification of dysarthria types among neurologists, residents in neurology and speech therapists. Eur Neurol. (2009) 61:295–300. 10.1159/000206855 19295217

[12] Rusz J Tykalova T Ramig LO Tripoliti E. Guidelines for speech recording and acoustic analyses in Dysarthrias of movement disorders. Mov Disord. (2021) 36:803–14. 10.1002/mds.28465 33373483

[13] Tourville JA Guenther FH. The DIVA model: a neural theory of speech acquisition and production. Lang Cogn Process. (2011) 26:952–81. 10.1080/01690960903498424 23667281 PMC3650855

[14] Ziegler W. Task-related factors in oral motor control: speech and oral diadochokinesis in dysarthria and apraxia of speech. Brain Lang. (2002) 80:556–75. 10.1006/brln.2001.2614 11896657

[15] Boersma, PWD. Praat: doing phonetics by computer [Computer program]. Available online at: www.praat.org (accessed February 20, 2023).

[16] Rusz J Cmejla R Ruzickova H Ruzicka E. Quantitative acoustic measurements for characterization of speech and voice disorders in early untreated Parkinson's disease. J Acoust Soc Am. (2011) 129:350–67. 10.1121/1.3514381 21303016

[17] Vogel AP Maruff P. Comparison of voice acquisition methodologies in speech research. Behav Res Methods. (2008) 40:982–7. 10.3758/BRM.40.4.982 19001389

[18] Goetz CG Tilley BC Shaftmann SR Stebbins GT Fahn S Martin PM. Movement disorder society- sponsored revision of the unified Parkinson's disease rating Scal (MDS-UPDRS): scale presentation and clinimetric testing results. Mov Disord. (2008) 23:2129–70. 10.1002/mds.22340 19025984

[19] Bürk K Mälzig U Wolf S Heck S Dimitriadis K Schmitz-Hübsch T. Comparison of three clinical rating scales in Friedreich ataxia (FRDA). Mov Disord. (2009) 24:1779–84. 10.1002/mds.22660 19562766

[20] Mariën P Ackermann H Adamaszek M Barwood CHS Beaton A Desmond J. Consensus paper: language and the cerebellum: an ongoing enigma. Cerebellum. (2014) 13:386–410. 10.1007/s12311-013-0540-5 24318484 PMC4090012

[21] Brendel B Synofzik M Ackermann H Lindig T Schölderle T Schöls L. Comparing speech characteristics in spinocerebellar ataxias type 3 and type 6 with Friedreich ataxia. J Neurol. (2015) 262:21–6. 10.1007/s00415-014-7511-8 25267338

[22] Spencer KA Amaral J Lansford K. Investigating perceptual subgroups in speakers with ataxic dysarthria: an auditory free classification approach. Am J Speech Lang Pathol. (2022) 23:1–11. 10.1044/2022_AJSLP-22-00159 36417768

[23] Bostan AC Strick PL. The basal ganglia and the cerebellum: nodes in an integrated network. Nat Rev Neurosci. (2018) 19:338–50. 10.1038/s41583-018-0002-7 29643480 PMC6503669

[24] Darley FL Aronson AE Brown JR. Motor Speech Disorders. Philadelphia: W B Saunders Company. (1975) p. 304.

[25] Darley FL Aronson AE Brown JR. Clusters of deviant speech dimensions in the dysarthrias. J Speech Hear Res. (1969) 12:462–96. 10.1044/jshr.1203.462 5811846

[26] Westbury JR Dembowski J. Articulatory kinematics of normal diadochokinetic performance. Annual Bulletin of the Research Institute of Logopedics and Phoniatrics, (1993) 27, 13–36.

[27] Mansur LL Radanovic M de Carvalho Araújo G Taquemori LY Greco LL. Teste de nomeação de Boston: desempenho de uma população de São Paulo. Pró-Fono Revista de Atualização Cientí*fica*. (2006) 18:13–20. 10.1590/S0104-56872006000100003 16625867

